# Quantifying Societal Burden of Radiation-Induced Cardiovascular Events in Breast Cancer Survivors

**DOI:** 10.3389/fonc.2022.869529

**Published:** 2022-04-12

**Authors:** Eva Kimpe, Amber Werbrouck, Mark De Ridder, Koen Putman

**Affiliations:** ^1^ Interuniversity Centre for Health Economics Research (I-CHER), Department of Public Health, Vrije Universiteit Brussel, Brussels, Belgium; ^2^ Department of Radiotherapy, Universitair Ziekenhuis Brussel, Vrije Universiteit Brussel, Brussels, Belgium

**Keywords:** radiotherapy, cardiotoxicity, breast cancer, survivorship, health economics

## Abstract

**Background and Purpose:**

Radiation-induced cardiotoxicity is an important health concern for clinicians during treatment of breast cancer (BC) patients. Underlying mechanisms are well-documented, whereas little is known about the societal impact of this long-term effect. This study aimed to quantify the additional burden of radiation-induced cardiovascular (CV) diseases in BC survivors.

**Materials and Methods:**

Conventional health economic modelling techniques were applied to estimate attributed CV-related costs and disutility in a hypothetical cohort of BC survivors. A situation in which radiotherapy caused an additional CV risk was compared with a situation in which this risk was not taken into account. Uncertainty was assessed *via* deterministic and probabilistic sensitivity analyses. Analyses were performed from a broad societal perspective up until 20 years after BC treatment.

**Results:**

Radiation-induced cardiotoxicity evokes a mean incremental cost of €275.10 per woman over a time horizon of 20 years after BC treatment. An additional decrement of 0.017 QALYs (per woman) might be expected when taking the radiation-induced cardiotoxic risk into account in BC survivors. Incremental costs and disutility increased with age. A scenario analysis showed that these results were more profound in women with more advanced staging.

**Conclusion:**

Our analyses suggest that with current radiation techniques, rather minor costs and disutility are to be expected from radiation-induced cardiotoxicity in BC survivors. The cost of past investments in order to achieve current mean heart dose (MHD) seems justified when considering the gains from cost and disutility reduction resulting from radiation-induced cardiovascular events. The question we might consider is whether future opportunity costs associated with investments on further technological advancements offset the expected marginal benefit from further reducing the MHD.

## 1 Introduction

Breast cancer (BC) is the most frequently diagnosed cancer type in women worldwide, accounting for more than one quarter of all newly diagnosed female cancers (1, 2). Early detection of breast cancer is enhanced by nationwide accessibility of screening programs for women at risk (3). As a result, breast cancer is diagnosed more frequently at an early stage, leading to a beneficial prognosis. Concurrently, advances in radiotherapy have led to a significant reduction in local recurrence and BC mortality (4). Radiation is administrated to over half of all BC patients (5), and therefore, it is likely that radiotherapy contributes to high survival rates. In developed countries, such as Belgium, the 5-year relative survival rate is around 91% (6).

However, radiation from radiotherapy is not limited to tumor tissue. Despite the use of state-of-the-art radiation techniques, healthy organs -such as the heart- are exposed to minor radiation, leading to inflammatory responses in this tissue (7). These radiation-induced cardiotoxic effects expose BC survivors to an increased long-term risk for cardiovascular (CV) diseases (7–9). These events may have an additional impact on the health-related quality of life (HRQoL), in particular as HRQoL might already have been influenced by BC treatment prior to a potential CV event (10). Additionally, CV events are associated with an increased CV-related mortality risk (11). A meta-analysis demonstrated that the rate ratio for CV mortality was 1.3 (SE 0.09; 2p=0.0007) in BC patients receiving RT compared to controls undergoing the same treatment without RT. Although this meta-analysis provided evidence on benefits from reduction in breast cancer mortality by RT during treatment, the researchers demonstrated the moderate increase in death from CV events in particular in the first and second decade after treatment (4). Other studies indicated similar results (12). However, these studies mainly addressed CV mortality. Although research indicated that the economic burden of cardiovascular diseases soars compared to the health expenditure for other diseases, estimated around 9% of total health care expenditure across European countries (13), the long-term health outcome and costs of cardiovascular morbidity in breast cancer survivors are less considered. The application of health economic evaluation techniques poses an opportunity in order to model these long-term CV effects as it “*provides a framework to make best use of clinical evidence through an organized consideration of the effects [ … ], health care costs, and other effects that are regarded as valuable*” (17). Formerly, health economics research in the field of oncology have largely focused on costs and effects related to primary BC treatment rather than exclusively on long-term CV mortality and morbidity (14–16).

Most of these health economic studies did not include long-term cardiotoxic costs and effects in their analysis. There is still a lack in the more precise quantification of the societal burden of radiation-induced CV diseases. Our study aims to quantify the additional burden of radiation-induced cardiovascular diseases in BC survivors.

## 2 Materials and Methods

### 2.1 Study Design and Perspective, Target Population and Setting

We employed decision-analytic modelling techniques in which data from various sources is combined to populate a theoretical model (17). We used two types of cohort models, a decision tree and Markov model, to estimate the proportion of patients that would encounter an event over the intended time horizon (i.e. cardiovascular events and/or death). During each cycle, these proportions are attributed to different health states which relate to costs and disutilities (18). The model simulates a cohort of 39-84 year old women diagnosed with stage 0-3 breast cancer, and who were eligible for curative primary BC treatment with or without radiotherapy. Since radiotherapy is not standard care for women with metastatic cancer (stage 4), they were excluded from the cohort. The administration of chemotherapy was left out because this would not differ between the evaluated alternatives, and therefore, would not influence the reported incremental result. Data originates from various sources (European and American studies, Belgian databases). Therefore, the model utmost applies to populations with similar female BC incidence rates, and adherence to the proposed treatment guidelines on primary BC treatment (19–22). A broad, societal perspective is adopted in this cost-utility analysis (CUA). In this perspective, the term “societal burden” refers to cost and consequences of diseases to society (17). In contrast to a health care payer perspective, a wider range of costs is included in this perspective. Typically, indirect costs generated outside the health care system are included in the analyses (17). Hence, direct medical health care costs (primary care, outpatient care, inpatient care, prehospital E&A care and pharmaceuticals) and indirect health care costs (production loss due to premature death, sick leave and permanent disability) were considered. The study protocol was approved by the ethical committee at the Universitair Ziekenhuis Brussel, Brussels, Belgium (B.U.N. 1432020000259). All analyses were performed in Microsoft Excel^®^ 2016.

### 2.2 Cycle Length, Time Horizon and Discount Rate

Cardiovascular events are typically emerging as a long-term effect from radiotherapy, starting within the first 5 years up until 30 years after primary treatment (23). A time horizon of 20 years after BC treatment was considered. The cycle length was determined at one year. In accordance to Belgian guidelines, discount rates of 3% on costs and 1.5% on outcomes were applied (24).

### 2.3 Model and Model Assumptions

In this study, we combined a decision tree and Markov model to model probabilities, expected costs and expected (dis)utilities in order to stratify the cohort based on their exposure to radiotherapy and analyse long-term radiation-induced cardiotoxic effects, respectively. A radiation-oncologist (MDR) was closely involved in the modelling process to validate the assumptions in both models to assure accordance with clinical practice.

#### 2.3.1 Model I: Primary Breast Cancer Treatment (Decision Tree)

**Figure 1:**
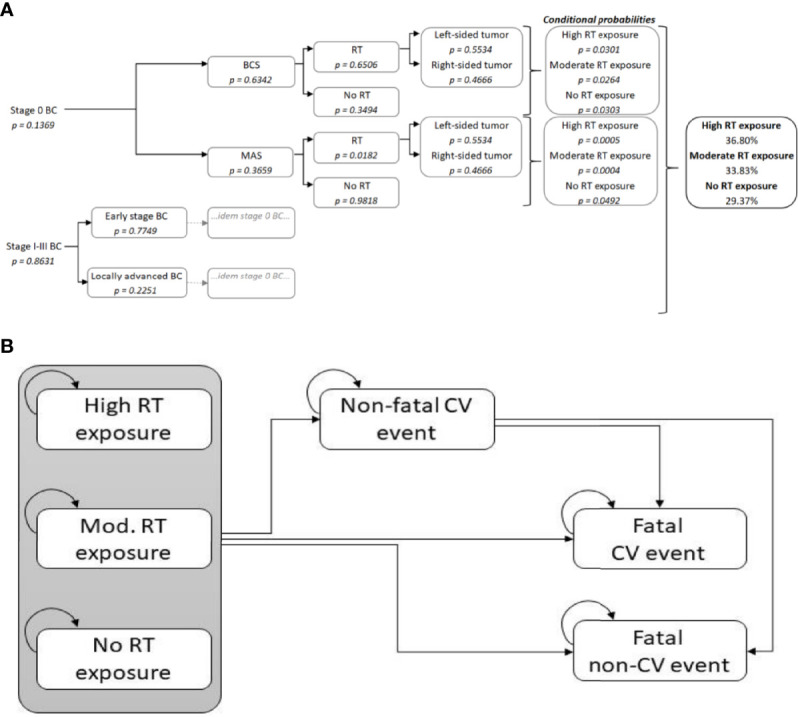
**(A)** Decision tree. The branches represent different primary breast cancer treatment pathways. When radiotherapy is administrated, this results in a radiation dose to the heart according to the laterality of the tumour (for further details see **Supplementary Material 1**), **(B)** Markov model. The boxes represent the six health states of the Markov model. During each yearly cycle, patients may remain in the same state, or transition to another state (represented by the arrows in the figure). BC, Breast Cancer; BCS, Breast Conserving Surgery; CV, Cardiovascular; MAS, Mastectomy; RT, Radiotherapy.

The decision tree represents different treatment pathways in primary BC patients (19–22). The different branches result in either receiving radiotherapy or not. For women who received radiotherapy, left- and right-sided tumors were assumed to lead to high or moderate radiation exposure, respectively. No radiation exposure was assumed for women who did not receive radiotherapy. The endpoints served as the starting point to stratify female BC patients in the initial states of the Markov model (**Figure 1A**, for details see **Supplementary Material 1, 2**).

#### 2.3.2 Model II: Long-Term Radiation-Induced Cardiotoxicity (Markov Model)

The initial states were defined as [1] ‘high radiotherapy exposure’, corresponding with a mean heart dose (MHD) of 3.6 Gy [2], ‘moderate radiotherapy exposure’, correlating with 1.9 Gy and [3] ‘no radiotherapy exposure’, assumed for women who did not receive radiotherapy (i.e. 0 Gy), and accounting for the laterality of the tumor. The respective MHD means (i.e. 3.6 Gy and 1.9 Gy) were based on a recent systematic review reporting studies published between January 2014 and September 2017. As a variety of RT techniques (e.g. 3D-CRT, step-and-shoot IMRT, rotational IMRT,…), with target doses between 39.9 Gy and 50.4 Gy were reported (25), we believed the assumed MHD correlated well with characteristics from our cohort.

Starting from the initial states, patients either stay in this state, or transfer to one of the three other states. Firstly, a patient may experience a non-fatal cardiovascular event. Secondly, the patient may also immediately die from the cardiovascular event. Lastly, the patient could also decease from other causes (i.e. fatal non-CV events). The two fatal event states were absorbing states, meaning that patients who entered one of the fatal states could not transition back to the other states (**Figure 1B**).

### 2.4 Comparators and Analytic Methods

#### 2.4.1 Comparators

In order to quantify the additional burden of radiation-induced CV diseases in BC survivors, we evaluated two alternatives: [1] a situation in which an increased radiation-induced cardiovascular risk was considered, and [2] a comparative situation in which the additional risk of radiotherapy was not taken into account (see **Supplementary Material 3**). This strategy was based on a methodology proposed by Mulliez et al. Their approach consisted of adding an excess risk to the traditional SCORE calculation for women who were exposed to radiotherapy (26).

#### 2.4.2 Analytical Methods

A theoretical cohort of 1,000 female BC survivors aged 39-84 years at diagnosis was assumed. Each age in the cohort was weighted according to Belgian age-specific incidence rates. Expected cost and quality adjusted life years (QALYs) for the two situations were calculated. Typically in a traditional health-economic evaluation, incremental costs and effects would be presented as cost-effectiveness ratios (ICERs). Since a negative health outcome (i.e. less QALYs) was expected in the situation in which the increased radiation-induced CV risk was considered, calculating ICERs was irrelevant. Therefore, results were presented as (mean) incremental costs and (mean) incremental QALYs per woman over a time horizon of 20 years after BC diagnosis.

In the deterministic base case analyses the model was ran based on the most likely assumptions and input parameters (27). Different deterministic scenario analyses were performed. In these scenarios we examined results within specific subgroups by stage and radiation strategy.

In the sensitivity analyses, base case assumptions and input variables were altered in order to assess the uncertainty in the model (27). In a deterministic one-way sensitivity analysis, the most influential parameters were determined by decreasing and increasing each variable separately by an arbitrary chosen proportion of 30% (i.e. 70% and 130% of the base case value). These results were visualized in Tornado diagrams for incremental costs and health outcomes separately. Finally, a probabilistic sensitivity analysis (PSA) was performed *via* Monte Carlo simulations. We ran 1,000 iterations to evaluate the uncertainty around the base case point estimates. Sampling was established by applying distributions which fitted characteristics of the parameters (**Table 1**).

**Table 1:** Model input parameters (probabilities, costs and utilities).

Parameter	Deterministic value	Distribution	Source
**Probabilities and SCORE input variables**			
Cohort stratification: high RT exposure	0.3680^*^		*Decision tree*
Cohort stratification: moderate RT exposure	0.3383^*^		*Decision tree*
Cohort stratification: no RT exposure	0.2937^*^		*Decision tree*
Mean heart dose: high RT exposure	3.6000	Log-normal	(25)
Mean heart dose: moderate RT exposure	1.9000		(25)
Mean heart dose: no RT exposure	0.0000		
Calculated ratio MHD high RT/MHD moderate RT	1.8947	Log-normal	
Excess risk ratio for cardiotoxicity	0.0410	Log-normal	(28)
Systolic blood pressure 40-64 years	116.0847	Normal	(29)
Systolic blood pressure 65+ years	136.2094	Normal	(29)
Total cholesterol 40-64 years	5.1832	Normal	(29)
Total cholesterol 65+ years	5.4144	Normal	(29)
Rate of smokers in a cohort of breast cancer survivors	0.0951	Beta	(30)
*Probability of fatal CV event in breast cancer survivors^+^*	*0.0427*	*Beta*	(31)
*Probability of non-fatal CV event in breast cancer survivors^+^*	*0.1175*	*Beta*	(31)
Calculated risk of non-fatal CV events/fatal CV events^+^	2.7515		
Probability of fatal CV event after non-fatal CV event	0.0115	Beta	(32)
Probability for fatal non-CV event in breast cancer survivors	0.0181	Beta	(31)
Work-related activity rate for women	0.6490	Gamma	(33)
Probability of permanent disability after non-fatal CV event	0.0394	Beta	(34)
Probability of temporarily sick leave after non-fatal CV event	0.9616		(35)
Mean sick leave days during first year after non-fatal CV event	48.0000	Gamma	(33)
Mean sick leave days in following years after non-fatal CV event	25.0000	Gamma	(33)
Costs**			
Annual wage for women aged 40-65 years, anno 2021	55,559.7500	Gamma	(36)
Daily wage for women aged 40-65 years, anno 2021	252.3000	Gamma	(36)
Primary care costs during first year after non-fatal CV event	54.1200	Gamma	(37)
Primary care costs in following years after non-fatal CV event	94.7100	Gamma	(37)
Outpatient care costs during first year after non-fatal CV event	196.3300	Gamma	(37)
Outpatient care costs in following years after non-fatal CV event	155.7800	Gamma	(37)
Prehospital A&E care costs after non-fatal and fatal CV event	455.6200	Gamma	(38)
Inpatient care costs after non-fatal CV event	4547.5100	Gamma	(39)
Pharmaceutical costs after non-fatal CV event	149.0882	Gamma	(40)
**Utilities (QALYs)**			
Utility for women in the initial state(s)	0.7700	Beta	(29)
*Disutility for chronic CV disorders in Vietnam°*	*0.1100*		(41)
*Quality of life in Vietnam (all ages)°*	*0.9100*	*Log-normal*	(41)
Calculated utility for women in the non-fatal CV event state	0.6769		

^*^Cohort stratification is based on age-specific incidence rates resulting from the decision tree (see **Supplementary Materials 1, 2**). The values presented in this table are mean values for all women in the modelled cohort (39-84 year).

^+^For details on all age-specific transition probabilities for non-fatal CV events and fatal CV events, see **Supplementary Material 4**.

A&E, Accident & Emergency; CV, Cardiovascular; MHD, Mean Heart Dose; RT, Radiotherapy; QALYs, Quality Adjusted Life Years.

** All costs are converted to and expressed in EUR (€, 2021).

° Relative disutility was calculated from a Vietnamese cohort with resembling characteristics. This proportional decrease was applied to Belgian female population to calculate ‘utility for women in the non-fatal CV event state’.

### 2.5 Study Parameters

In this model-based analysis various sources were used to populate the two models (**Table 1**). Population-based national data, from the Belgian Cancer Registry (2017), and secondary data retrieved from the literature were used to populate the decision tree (for details see **Supplementary Material 1, 2**). In the Markov model we included secondary data from literature, and data from the Belgian Health Examination Survey, 2018 (29). The risk of cardiovascular mortality was estimated *via* the SCORE equations (42). An excess risk of 4.1% per Gy MHD was considered to account for the radiation effect (26). Detailed information on transition probabilities for fatal and non-fatal CV events is provided in **Supplementary Material 4**. The input variables for the SCORE calculation (i.e. systolic blood pressure and cholesterol) were based on age-specific mean values for Belgian females (29). Smoking status was added as a weighted mean of smokers in a population of BC survivors (30). We used event rates for fatal and non-fatal CV events from a Dutch breast cancer cohort study to calculate the relative risk (RR) for non-fatal CV events, resulting in a RR of 2.75 non-fatal CV events for each fatal CV event (31). Concerning the transition from the non-fatal CV event state to the fatal CV event state, event rates from the EUROASPIRE were applied (32). We established the probability for a fatal non-CV event by excluding cardiovascular event rates from all-cause mortality rates. The latter was retrieved from the same Dutch cohort study that was used to extract non-fatal CV events (31). All probabilities were recalculated to annual transition probabilities (17).

### 2.6 Costs

An overview of costs is given in **Table 1**. In the analysis of costs, only costs as a result of radiation-induced cardiotoxicity were considered. Costs related to the primary BC treatment were not included, as these were nullified between both alternatives. Direct costs are retrieved from various Belgian and European sources (37–40). Assumptions are based on clinical practice guidelines and -if deemed necessary- validated through expert opinion (MDR). Costs resulting from productivity loss (indirect costs) were based on Belgian wages (33, 36), and calculated using the human capital approach pertaining temporarily or permanent disability and premature death (17). All costs were converted to current prices (2021 euros) with the CCEMPG-EPPI-Centre Cost Converter (43).

### 2.7 Health Outcomes

QALYs are the preferred measure of health outcome in CUA (17). As with costs, we assumed that the benefit of prolonged survival from radiotherapy would be equal in the two comparative situations as the cohorts received the same primary breast cancer treatment. Research indicates HRQoL in long-term BC survivors is not significantly different from HRQoL in age-matched women in general population (44, 45). Therefore, we used HRQoL from a national survey for women in the initial states (29). The relative disutility for chronic cardiovascular disorders was calculated from a recent comprehensive systematic review (41), which comprised data from a cohort with resembling characteristics (46). The relative disutility was calculated as proportional decrease in HRQoL in the reference population of this study (46). Then, this proportional decrease was applied to the HRQoL in Belgian females (29) and served as basis for the utility of women experiencing a non-fatal CV event. Utilities are summarized in **Table 1**.

## 3 Results

### 3.1 Base Case Analyses

**Figure 2:**
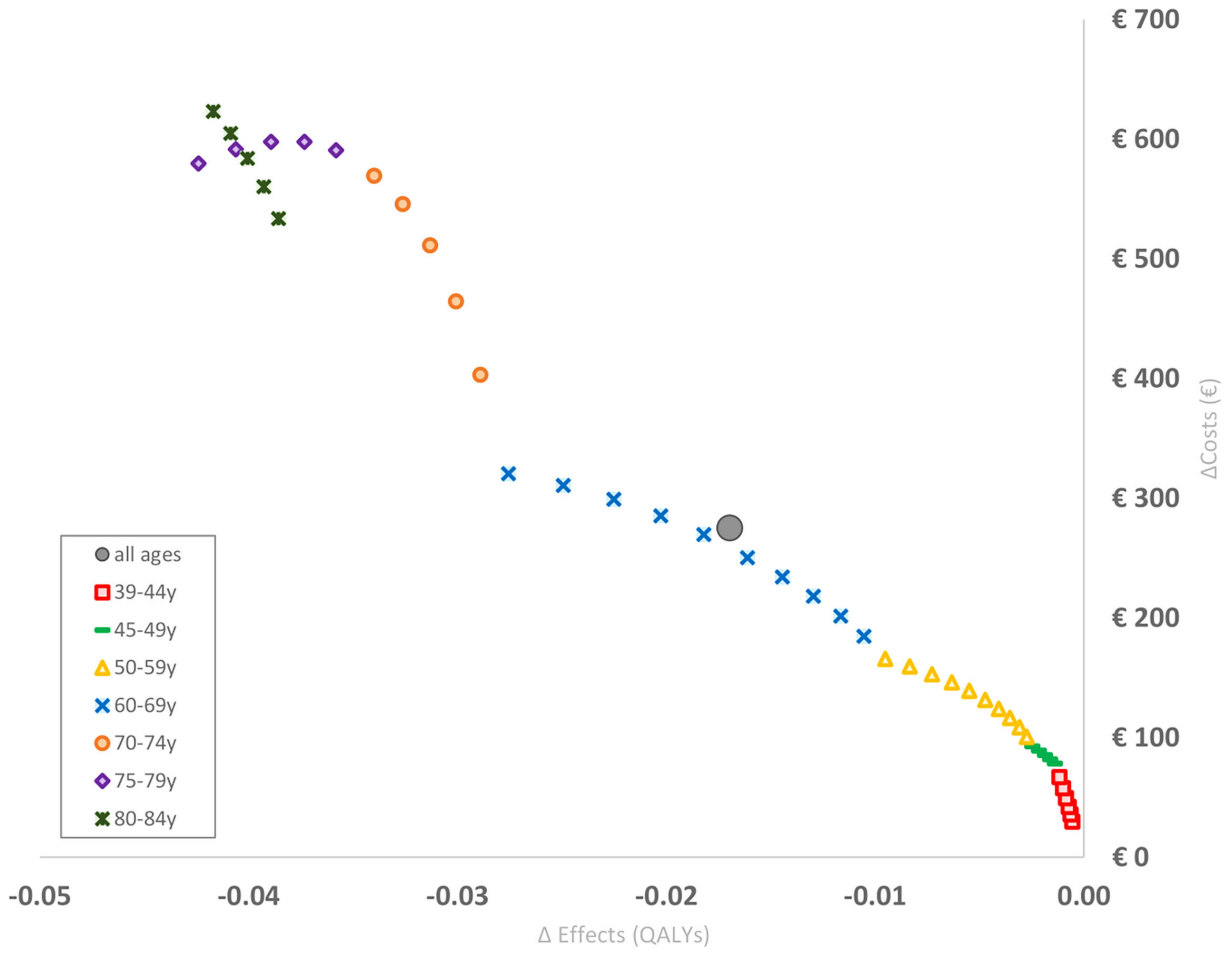
Base case analysis. Each small point estimate represents the incremental cost and disutlity for a respective age (unweighted for age-specific incidence rates) over a time horizon of 20 years after BC diagnosis, and shows the increasing trend with aging. The large point estimate indicates the mean incremental cost and disutility for all women in the cohort (i.e. Δ costs , €275.10 and Δ disutility , 0.017 QALYs). BC, Breast Cancer; QALYs, Quality Adjusted Life Years; y, years.

Baseline results are presented in **Table 2**, showing expected numbers of non-fatal and fatal CV events per 1,000 women over a time horizon of 20 years after BC treatment. Overall, the situation in which cardiotoxicity was taken into account resulted in more than four additional non-fatal CV events and more than two additional fatal CV events. These extra cases resulted in an average incremental cost of €275.10 per woman, and an average incremental utility decrement of 0.017 QALYs per woman. The base case analysis revealed that increasing age is associated with accumulating incremental costs and decreasing incremental QALYs (**Figure 2**). Direct costs appeared to be the main contributors in total costs, being thirteen times higher than indirect costs (€254.16 vs. €20.94).

**Table 2:** Baseline results in a total cohort of 1,000 female breast cancer survivors^1^.

Age at diagnosis	% of the population^c^	Expected number of non-fatal CV events			Expected number of fatal CV events		
		RT risk taken into account^a^	RT risk not taken into account^b^	Δ	RT risk taken into account^a^	RT risk not taken into account^b^	Δ
39-44	6.36%	0.73	0.70	+0.03	0.30	0.29	+0.01
45-49	9.40%	2.08	2.00	+0.08	0.89	0.85	+0.03
50-54	12.39%	6.39	6.15	+0.24	2.61	2.51	+0.10
55-59	12.67%	11.93	11.49	+0.44	4.95	4.77	+0.18
60-64	14.10%	23.71	22.86	+0.84	9.65	9.32	+0.32
65-69	14.26%	36.58	35.36	+1.21	15.11	14.64	+0.46
70-74	12.31%	53.81	52.81	+1.00	18.26	17.77	+0.48
75-79	9.84%	57.91	57.40	+0.51	18.41	18.02	+0.39
80-84	8.67%	61.43	61.46	-0.04	18.22	17.90	+0.31
**Total (all ages)**	**100%**	**254.56**	**250.24**	**+4.32**	**88.39**	**86.09**	**+2.30**

^1^Expected number of non-fatal CV events and fatal CV events 20 years after breast cancer diagnosis, comparing the situation in which radiation-induced cardiotoxicity is taken into account (columns a) and the situation in which no additional risk of radiation-induced cardiotoxicity is considered (columns b) during analysis. Results are presented for a total cohort of 1,000 women in which the events are weighted according to their age-specific incidence rates (column c).

CV, Cardiovascular; RT, Radiotherapy.

### 3.2 Deterministic Scenario Analyses

**Figure 3:**
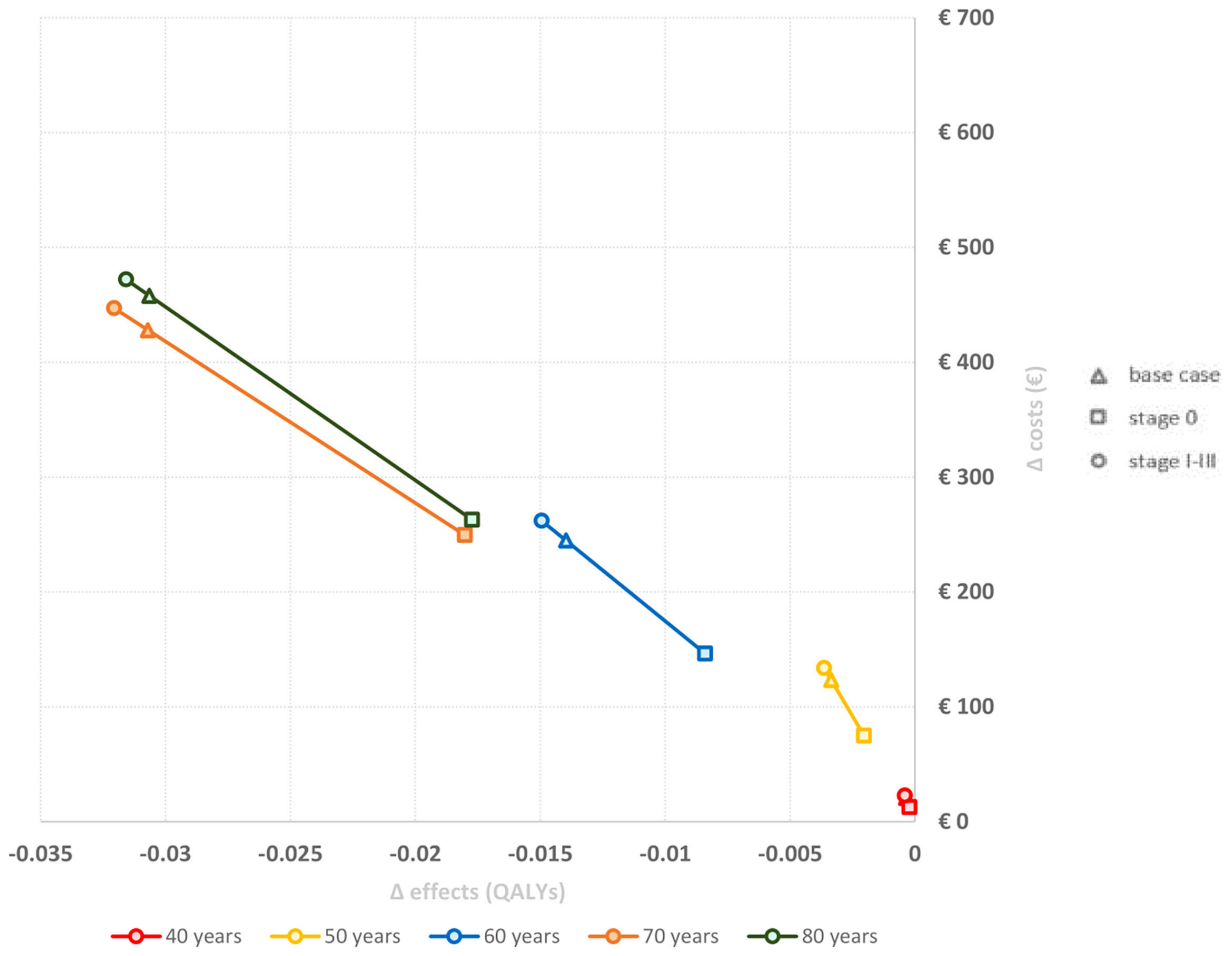
Deterministic scenario analysis: Incremental costs and disutility by stage. The proportions of the start cohort (decision tree) were set to 0% or 100% in order to exclude either stage 0 or stage 1-3 from analysis. This resulted in various initial state cohort stratifications, which is indicated through different symbols. Results are presented as incremental costs and QALYs per woman for four different age groups (40 years, 50 years, 60 years, 70 years and 80 years). BC, Breast Cancer; QALYs, Quality Adjusted Life Years; y, years.

In the base case analysis, all women eligible for radiotherapy administration after BC diagnosis were considered, regardless of stage (stage 0-3) and RT administration (with or without radiotherapy). **Figure 3** represents a first series of scenario analyses, which demonstrate the effect of the stage at time of diagnosis. For all ages, a diagnosis of breast cancer at an earlier stage resulted in lower costs and higher QALYs compared to more advanced breast cancers. Radiotherapy is more likely to be omitted in stage 0 BC, resulting in less women in the high and moderate radiotherapy exposure groups, and therefore, explaining this effect. Furthermore, the relative difference became more profound in women diagnosed at older age. A second scenario analysis, showed that base case incremental costs and disutility were underestimated if only women who effectively received RT were included. Incremental costs increased with €109.53, while incremental QALYs aggravated with -0.0067 compared to the base case (Δ costs: €384.63 per woman, Δ QALYs: -0.024 per woman).

### 3.3 Sensitivity Analyses

**Figure 4:**
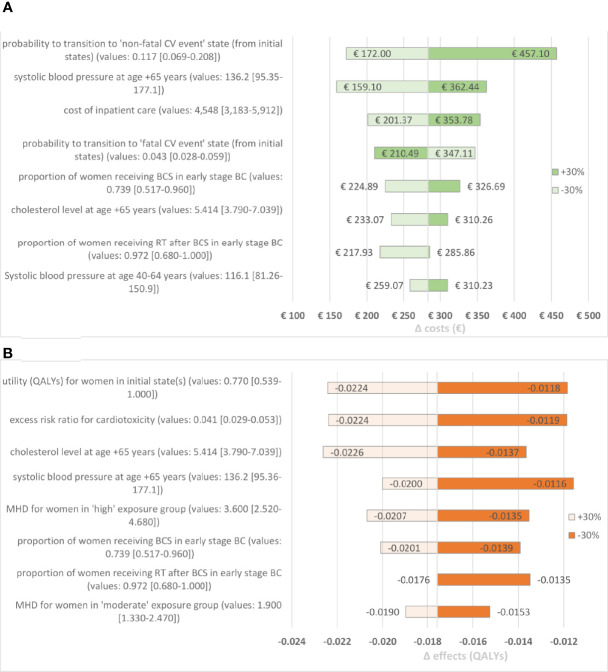
**(A)** Tornado diagram for costs. This diagram represent the impact on incremental costs when setting the parameter to 70% and 130% of the base case value. The 8 most influential parameters are presented. **(B)** Tornado diagram for QALYs. This diagram represent the impact on incremental QALYs when setting the parameter to 70% and 130% of the base case value. The 8 most influential parameters are presented. BC, Breast Cancer; BCS, Breast Conserving Surgery; CV, Cardiovascular; MAS, Mastectomy; MHD, Mean Heart Dose; QALYs, Quality Adjusted Life Year.

One-way sensitivity analysis identified the following parameters as most influential on incremental costs: (1) probability to transition to the ‘non-fatal CV event’ state from initial state, (2) systolic blood pressure for women aged 65+ years, (3) cost of inpatient care (**Figure 4A**). For incremental QALYs, most affecting parameters were (1) utility for women in initial state, (2) excess risk ratio for cardiotoxicity, (3) cholesterol level for women aged 65+ years (**Figure 4B**).

In the PSA, all iterations -as expected- resulted in higher costs and less QALYs. Consequently, if an additional radiation-induced cardiovascular risk is taken into account in BC survivors in order to estimate long-term costs and outcomes, this will always result in lower health outcomes and higher costs. The analysis showed that the mean value of incremental cost over all iterations was €131 (minimum) and €2,678 (maximum) per woman over a time horizon of 20 years (σ² = 54,292.67; σ= 233.01), respectively. Similarly, incremental disutility among the iterations of the average value of all iterations varied between a minimum of -2.727x10^-4^ QALYs and maximum of -0.564 per woman (σ² = 2.803^-3^; σ= 0.053).

## 4 Discussion

Our study focused on the incremental costs and effects attributed to radiation-induced cardiotoxicity. The analysis showed that radiotherapy is associated with four additional non-fatal and two additional fatal CV events in a cohort of 1,000 women over a time horizon of 20 years. In effect, the impact of taking radiation-induced cardiotoxic effects in account remains limited to an incremental cost of €275.10 per woman and incremental utility decrement of 0.017 per woman over 20 years.

Lundkvist et al. performed a CUA of proton beam therapy compared to conventional radiotherapy. As in our study, they choose non-fatal and fatal CV events as outcome measure in their Markov model. They expected 11.8 non-fatal CV events, and 3.3 fatal CV events per 100 55-year old women over a time horizon of 23 years (47). In our study, we observed, respectively, 9.6 events and 1.6 events in this age group over 20 years. Hence, the analyses produced a close match, supporting the assumptions in our model.

In our analysis, incremental costs increased with age, resulting in highest costs for older age groups. In effect, adjusting for age-specific incidence rates from different countries might reflect in higher costs for younger women. Direct costs were the main cost contributor in total costs. This is in line with results reported by Wilkins et al. who stated that direct health care costs contributed for over half of total CV-related costs in Europe (48). In our study the explanation lies in the increasing risk for non-fatal CV events at older age, accumulating in high inpatient care costs. The comparison between Lundkvist et al. and our results demonstrated earlier that the use of different data sources and extrapolating results over longer time horizons might reflect in -although small- differences between studies. As in our study, it remains a challenge to correctly extrapolate events over a long time horizon.

Our study is subject to several limitations. Firstly, it is important to notify that cardiotoxicity is not solely generated by radiation during BC treatment. Many studies in the field of cardio-oncology use the term ‘treatment-induced cardiotoxicity’ (49–51). Therefore, it could be considered a narrow approach to isolate radiotherapy from other cardiotoxic treatment factors (e.g. anthracyclines and HER-2 antagonists) (50, 52). On the other hand, in recent years the use of statins was widely introduced in order to reduce cardiovascular risk (53). Secondly, we assumed that a time horizon of 20 years would suffice in order to quantify radiation-induced cardiotoxicity since a reference paper in this field suggested an increased risk starting five years up until three decades after treatment (23). Hence, one might discuss a lifetime horizon in this matter is more appropriate. Especially for younger age groups in our model, the probability of an event after the analytic horizon of 20 years is plausible. In analogy to the EUROACTION study (54), we foresaw difficulties in modelling events in younger women because various covariables may intervene in longer time horizons. Especially in models concerning CV diseases, the probability of an event depends on several dependent parameters. Briggs et al. have suggested to introduce covariance in probabilistic analyses *via* Cholesky decomposition. Unfortunately, in most cases information is lacking regarding the underlying covariance structure to incorporate interdependency in the analyses. Therefore, it is common to treat all parameters as independent (55). We used numerous studies and data sources as vehicles for our economic evaluation. The possibility to combine data from different sources poses major opportunities for health economics, but this also increases the degree of uncertainty in the model (17). Thirdly, it is noteworthy that -though we strived to incorporate as much relevant evidence- some parameters were not included, possibly leading to an oversimplification of some aspects in the model. For example, the SCORE equations were updated in June 2021 allowing to add even more parameters to the prediction such as HDL-cholesterol and pre-existing diabetes mellitus (56). Also, the analyses of Darby et al. suggested that the risk for radiation-induced cardiotoxicity increased over time (23) whereas in our analyses the risk remains 4.1% per Gy. However, we employed the method which was proposed by Mulliez et al. to make estimations on the cardiovascular risk in breast cancer survivors (26). As argued by Briggs et al. highly complex model structures are not always more appropriate and simplified assumptions allow researchers to keep the model manageable (55). Finally, the authors would like to emphasize that results from health economic modelling need to be considered in the light of a specific context, meaning the cohort models contain various sources of evidence (e.g. transition probabilities, cost resources and health outcome measures) (17). Generalizability is only recommended to populations with a resembling context. Moreover, we intended to adopt a societal perspective, though we limited our cost resources to direct medical costs and indirect costs due to production loss. As with most CUA, it could be argued that we omitted some relevant cost resources (such as transportation costs, domestic help,…) or even costs in other sectors outside health care.

To our knowledge, our study is the first study which uses health economic modelling techniques to estimate the long-term costs and effects of radiation-induced cardiotoxicity. The use of these techniques can serve as a framework for the evaluation of the impact of a health risk on a population (57). Therefore, our results are applicable to future health economic research in the field of cardio-oncology. A noteworthy element in our analyses is the extent of MHD as influential parameter. Several studies revealed that heart doses were extensively higher in the past compared to current MHD (58, 59). Due to the awareness of long-term comorbidities in BC patients, substantial improvements on radiation therapy technology and protocols led to a reduction of the radiation dose to the heart (25). To date, research emphasizes the importance of continuing to invest in technological advances in heart-sparing techniques in order to further reduce the MHD (60). Our analyses showed that current radiation doses already evoke negligible incremental costs and disutility at population level. These findings emphasize the importance of previous technological innovations. Incremental costs and disutility must have been significantly higher in the past since MHD decreased substantially over the past decades. Hence, the cost of past investments in order to achieve current radiation dosages appears to be justified when considering the gains from cost and disutility reduction resulting from cardiotoxicity in BC survivors.

In conclusion, our analysis quantified more in detail the impact of radiation-induced cardiotoxicity in BC survivors. Our findings demonstrated that with current radiation techniques, minor overall mean incremental costs and disutility are to be expected over a time horizon of 20 years after primary BC treatment. Major research investments have been made in order to substantially decrease the MHD. Our findings highlight the importance of these investments when considered against the reduced costs and disutility from cardiovascular events as a result from these innovations. On the other hand, our analyses might also open a debate on potentially high opportunity costs of future investments since the expected marginal gains from further reducing the MHD may no longer outweigh the research budget.

## Data Availability Statement

All data referring to parameters is available in the article or supplementary material. Further inquiries should be directed to the corresponding author.

## Author Contributions

EK: primary investigator and author (first draft preparation). AW: health economist and author (review and editing). MR: radiation oncologist (medical expertise) and senior author (review and editing). KP: health economist, principal investigator and senior author (review and editing). All the authors contributed to and agreed with the submitted manuscript.

## Funding

This research was established within the Strategic Research Programme (zwaartepunt, SRP 53, 2019-2024) ‘Societal Benefit of Markerless Stereotactic Body Radiotherapy: a Statistical Support based on Quantitative Imaging’ (SMARTQI of the Vrije Universiteit Brussel).

## Conflict of Interest

The authors declare that the research was conducted in the absence of any commercial or financial relationships that could be construed as a potential conflict of interest.

## Publisher’s Note

All claims expressed in this article are solely those of the authors and do not necessarily represent those of their affiliated organizations, or those of the publisher, the editors and the reviewers. Any product that may be evaluated in this article, or claim that may be made by its manufacturer, is not guaranteed or endorsed by the publisher.
