# Supplementary material for: Quantifying Societal Burden of Radiation-Induced Cardiovascular Events in Breast Cancer Survivors
Source: Front Oncol. 2022 Apr 12;12:869529. doi: 10.3389/fonc.2022.869529 (PMC9039176; doi:10.3389/fonc.2022.869529)
Supplement: Supplementary file 1 [file DataSheet_1.doc]

Supplementary Material

# Supplementary material 1 – Table 3

| **Supplementary material 1**  Table 3. Model input parameters (decision tree) | | | | |
| --- | --- | --- | --- | --- |
| **Parameter** | **Model value** | **N** | **Rates/events** | **Source** |
| **Stage 0 BC** |  |  |  |  |
| Incidence rate of stage 0 BC in Belgium (39-84 year), anno 2017 | 0.1369 | 10,063 | 1,377 | [1] |
| Rate of BCS in stage 0 BC in Belgium (39-84 year), anno 2017 | 0.6342 | 1,386 | 879 | [1] |
| Rate of RT administration after BCS in stage 0 BC in the USA | 0.6506 | 100,371 | 65,301 | [2] |
| RT of no RT administration after BCS in stage 0 BC in the USA | 0.3494 | 100,371 | 35,070 | [2] |
| Rate of MAS in stage 0 BC in Belgium (39-84 year), anno 2017 | 0.3658 | 1,386 | 507 | [1] |
| Rate of RT administration after MAS in stage 0 BC in the USA | 0.0182 | 770 | 14 | [3] |
| Rate of no RT administration after MAS in stage 0 BC in the USA | 0.9818 | 770 | 756 | [3] |
| Rate of left-sided tumours in stage 0 BC in Belgium, anno 2017 | 0.5334 | 1,573 | 839 | [1] |
| Rate of right-sided tumours in stage 0 BC in Belgium, anno 2017 | 0.4666 | 1,573 | 734 | [1] |
| **Stage 1-3 BC** |  |  |  |  |
| Incidence rate of stage 1-3 BC in Belgium, anno 2017 | 0.8631 | 10,063 | 8,685 | [1] |
| **Early stage BC** |  |  |  |  |
| Incidence rate of early stage BC in Belgium (39-84 year), anno 2017 | 0.7749 | 8,685 | 6,730 | [1] |
| Rate of BCS in early stage BC in Belgium (39-84 year), anno 2017 | 0.7386 | 6,889 | 5,088 | [1] |
| Rate of RT administration after BCS in early stage BC in the Netherlands | 0.9720 | 22,361 | 21,734 | [4] |
| Rate of no RT administration after BCS in early stage BC in the Netherlands | 0.0280 | 22,361 | 627 | [4] |
| Rate of MAS in early stage BC in Belgium (39-84 year), anno 2017 | 0.2614 | 6,889 | 1,801 | [1] |
| Rate of RT administration after MAS in early stage BC in the Netherlands | 0.1231 | 17,646 | 2,173 | [4] |
| Rate of no RT administration after MAS in early stage BC in the Netherlands | 0.8769 | 17,646 | 15,473 | [4] |
| Rate of left-sided tumours in early stage BC in Belgium, anno 2017 | 0.5201 | 7,387 | 3,842 | [1] |
| Rate of right-sided tumours in early stage BC in Belgium, anno 2017 | 0.4799 | 7,387 | 3,545 | [1] |
| **Locally advanced BC** |  |  |  |  |
| Incidence rate of locally advanced BC in Belgium (39-84 year), anno 2017 | 0.2251 | 8,685 | 1,955 | [1] |
| Rate of BCS in locally advanced BC in Belgium (39-84 year), anno 2017 | 0.4544 | 581 | 264 | [1] |
| Rate of RT administration after BCS in locally advanced BC in the USA | 0.8695 | 2,927 | 2,545 | [5] |
| Rate of no RT administration after BCS in locally advanced BC in the USA | 0.1305 | 2,927 | 382 | [5] |
| Rate of MAS in locally advanced BC in Belgium (39-84 year), anno 2017 | 0.5456 | 581 | 317 | [1] |
| Rate of RT administration after MAS in locally advanced BC in the USA | 0.6651 | 16,502 | 10,976 | [5] |
| Rate of no RT administration after MAS in locally advanced BC in the USA | 0.3349 | 16,502 | 5,526 | [5] |
| Rate of left-sided tumours in locally advanced BC in Belgium, anno 2017 | 0.5194 | 2,268 | 1,178 | [1] |
| Rate of right-sided tumours in locally advanced BC in Belgium, anno 2017 | 0.4806 | 2,268 | 1,090 | [1] |
| *Abbreviations: BC = Breast Cancer ; BCS = Breast Conserving Surgery ; MAS = Mastectomy ; RT = Radiotherapy* | | | | |

## References supplementary table 1

1. Belgian Cancer Registry. (2017) Requested dataset 2017. Brussels, Belgium: Belgian Cancer Registry.

2. Giannakeas V, Sopik V, Narod SA. Association of radiotherapy with survival in women treated for ductal carcinoma in situ with lumpectomy or mastectomy. JAMA Netw Open. 2018;1(4):e181100. doi: 10.1001/jamanetworkopen.2018.1100.

3. Zujewski JA, Harlan LC, Morrell DM, Stevens JL. Ductal carcinoma in situ: trends in treatment over time in the US. Breast Cancer Res Treat. 2011;127:251–7. doi: 10.1007/s10549-010-1198-z.

4. van Maaren MC, de Munck L, de Bock GH, Jobsen JJ, van Dalen T, Linn SC, et al. 10 year survival after breast-conserving surgery plus radiotherapy compared with mastectomy in early breast cancer in the Netherlands: a population-based study. Lancet Oncol. 2016;17(8):1158–70. doi: 10.1016/S1470-2045(16)30067-5.

5. Mazor A, Mateo A, Demora L, Sigurdson ER, Handorf E, Daly JM, et al. Breast conservation versus mastectomy in patients with T3 breast cancers (>5 cm): An analysis of 37.268 patients from the National Cancer Database. Breast Cancer Res Treat. 2019;173(2):301–11. doi: 10.1007/s10549-018-5007-4.

# Supplementary material 2 – Figure 5

| **Supplementary material 2** Overview decision tree: Conditional probabilities for all women aged |
| --- |


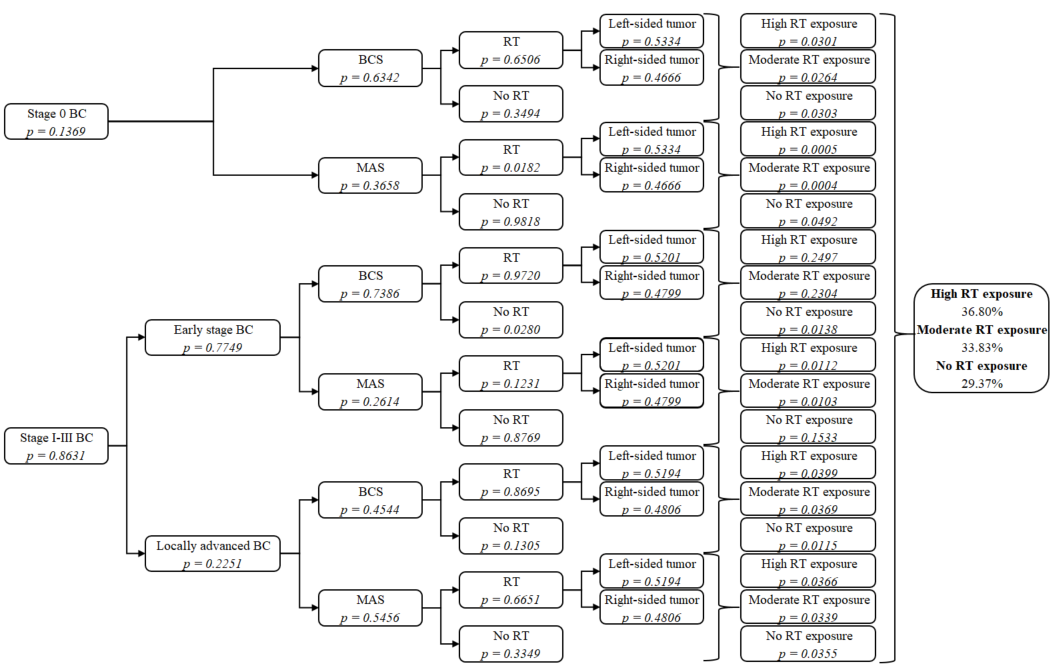


**Figure 5.** Decision tree. The decision tree precedes the Markov model and represents the conditional probabilities of breast cancer treatment. The model starts with a cohort of breast cancer patients (n = 100%) which are divided in stage 0 and stage 1-3 according to Belgian incidence rates in 2017, 13.69% and 86.31% for all women aged 39-84 years, respectively. However, for all calculations, these incidence rates were further adjusted to age-specific incidence rates. This was done in order to avoid an over- or under assumption of stages, and the corresponding treatment pathway, in specific age groups (e.g. stage 0 is more frequently diagnosed in younger women whereas stage 1-3 has higher incidence rates in older women).
Abbreviations: BC = Breast Cancer ; BCS = Breast Conserving Surgery ; MAS = Mastectomy ; RT = Radiotherapy

# Supplementary material 3 – Figure 6

**Supplementary material 3**Overview of the comparative analysis subject of the model


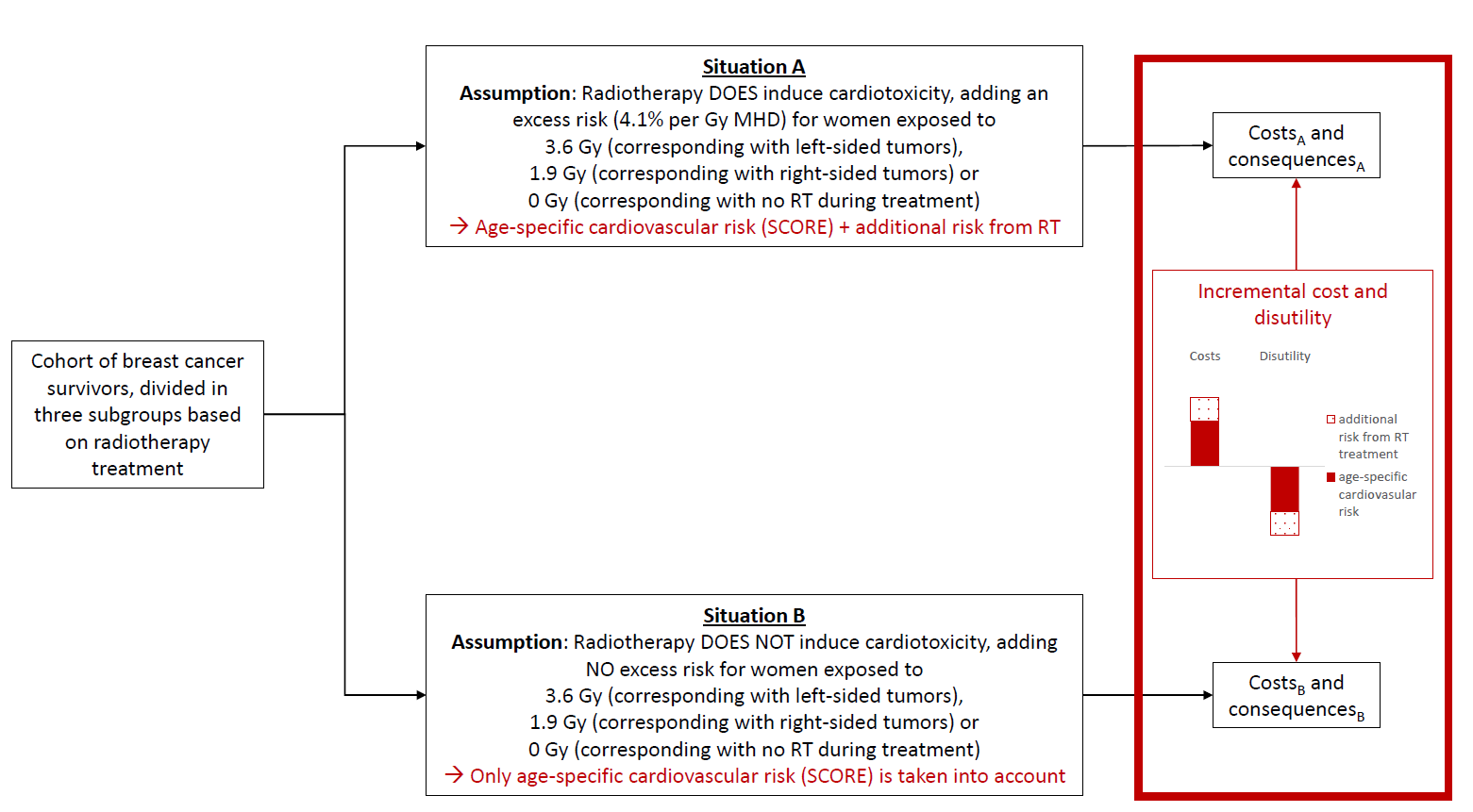


**Figure 6.** Comparative analysis. An overview is given of the two situations compared in this model. In the first situation (A) an addition risk for radiotherapy exposure (according to women receiving treatment for left- and right sided tumors, or receiving no radiotherapy during breast cancer treatment) was added to age-specific cardiovascular risk, based on SCORE equation. In the second situation (B), this risk was not taken into account, meaning that only reference age-specific cardiovascular risk (SCORE) was calculated. The analyses aimed on calculating the difference (i.e. incremental costs and disutility) between these situations in order to specifically quantify the burden from radiation-induced cardiotoxicity. To be clear, the benefit from radiotherapy on cancer survival and mortality was not considered directly since it was assumed this would be the same for both situations (and therefore would be nullified during analyses).

# Supplementary material 4 – Table 4

| **Supplementary material 4** Table 4. Detailed overview of cardiovascular mortality and morbidity transition probabilities | | |
| --- | --- | --- |
| **SCORE mortality prediction**^[[1]](#endnote-1)^ **: Transition probability from initial state to fatal CV event** | | |
| Age cohort: **40-49 year – non-smoker** | **Estimated 10-year transition probability** | **Estimated 1-year transition probability** |
| High radiotherapy exposure (3.6 Gy) – non-smoker | 0.0004 | 0.0000 |
| Moderate radiotherapy exposure (1.9 Gy) – non smoker | 0.0004 | 0.0000 |
| No radiotherapy exposure (0 Gy) – non smoker | 0.0004 | 0.0000 |
| Age cohort: **40-49 year – smoker** | **Estimated 10-year transition probability** | **Estimated 1-year transition probability** |
| High radiotherapy exposure (3.6 Gy) – smoker | 0.0008 | 0.0001 |
| Moderate radiotherapy exposure (1.9 Gy) – smoker | 0.0008 | 0.0001 |
| No radiotherapy exposure (0 Gy) – smoker | 0.0007 | 0.0001 |
| Age cohort: **50-59 year – non-smoker** | **Estimated 10-year transition probability** | **Estimated 1-year transition probability** |
| High radiotherapy exposure (3.6 Gy) – non-smoker | 0.0024 | 0.0002 |
| Moderate radiotherapy exposure (1.9 Gy) – non smoker | 0.0024 | 0.0002 |
| No radiotherapy exposure (0 Gy) – non smoker | 0.0022 | 0.0002 |
| Age cohort: **50-59 year – smoker** | **Estimated 10-year transition probability** | **Estimated 1-year transition probability** |
| High radiotherapy exposure (3.6 Gy) – smoker | 0.0046 | 0.0005 |
| Moderate radiotherapy exposure (1.9 Gy) – smoker | 0.0045 | 0.0004 |
| No radiotherapy exposure (0 Gy) – smoker | 0.0043 | 0.0004 |

| Age cohort: **60-69 year – non-smoker** | **Estimated 10-year transition probability** | **Estimated 1-year transition probability** |
| --- | --- | --- |
| High radiotherapy exposure (3.6 Gy) – non-smoker | 0.0091 | 0.0009 |
| Moderate radiotherapy exposure (1.9 Gy) – non smoker | 0.0088 | 0.0009 |
| No radiotherapy exposure (0 Gy) – non smoker | 0.0085 | 0.0008 |
| Age cohort: **60-69 year – smoker** | **Estimated 10-year transition probability** | **Estimated 1-year transition probability** |
| High radiotherapy exposure (3.6 Gy) – smoker | 0.0178 | 0.0018 |
| Moderate radiotherapy exposure (1.9 Gy) – smoker | 0.0172 | 0.0017 |
| No radiotherapy exposure (0 Gy) – smoker | 0.0165 | 0.0017 |
| Age cohort: **70-79 year – non-smoker** | **Estimated 10-year transition probability** | **Estimated 1-year transition probability** |
| High radiotherapy exposure (3.6 Gy) – non-smoker | 0.0411 | 0.0042 |
| Moderate radiotherapy exposure (1.9 Gy) – non smoker | 0.0398 | 0.0041 |
| No radiotherapy exposure (0 Gy) – non smoker | 0.0383 | 0.0039 |
| Age cohort: **70-79 year – smoker** | **Estimated 10-year transition probability** | **Estimated 1-year transition probability** |
| High radiotherapy exposure (3.6 Gy) – smoker | 0.0797 | 0.0083 |
| Moderate radiotherapy exposure (1.9 Gy) – smoker | 0.0771 | 0.0080 |
| No radiotherapy exposure (0 Gy) – smoker | 0.0742 | 0.0077 |
| Age cohort: **80-89 year – non-smoker** | **Estimated 10-year transition probability** | **Estimated 1-year transition probability** |
| High radiotherapy exposure (3.6 Gy) – non-smoker | 0.1003 | 0.0105 |
| Moderate radiotherapy exposure (1.9 Gy) – non smoker | 0.0972 | 0.0102 |
| No radiotherapy exposure (0 Gy) – non smoker | 0.0938 | 0.0098 |

| Age cohort: **80-89 year – smoker** | **Estimated 10-year transition probability** | **Estimated 1-year transition probability** |
| --- | --- | --- |
| High radiotherapy exposure (3.6 Gy) – smoker | 0.1918 | 0.0211 |
| Moderate radiotherapy exposure (1.9 Gy) – smoker | 0.1857 | 0.0203 |
| No radiotherapy exposure (0 Gy) – smoker | 0.1789 | 0.0195 |
| Age cohort: **90-99 year – non-smoker** | **Estimated 10-year transition probability** | **Estimated 1-year transition probability** |
| High radiotherapy exposure (3.6 Gy) – non-smoker | 0.2126 | 0.0236 |
| Moderate radiotherapy exposure (1.9 Gy) – non smoker | 0.2062 | 0.0228 |
| No radiotherapy exposure (0 Gy) – non smoker | 0.1991 | 0.0220 |
| Age cohort: **90-99 year – smoker** | **Estimated 10-year transition probability** | **Estimated 1-year transition probability** |
| High radiotherapy exposure (3.6 Gy) – smoker | 0.3961 | 0.0492 |
| Moderate radiotherapy exposure (1.9 Gy) – smoker | 0.3837 | 0.0472 |
| No radiotherapy exposure (0 Gy) – smoker | 0.3698 | 0.0451 |
| Age cohort: **100+ year – non-smoker** | **Estimated 10-year transition probability** | **Estimated 1-year transition probability** |
| High radiotherapy exposure (3.6 Gy) – non-smoker | 0.4005 | 0.0499 |
| Moderate radiotherapy exposure (1.9 Gy) – non smoker | 0.3886 | 0.0480 |
| No radiotherapy exposure (0 Gy) – non smoker | 0.3752 | 0.0459 |
| Age cohort: **100+ year – smoker** | **Estimated 10-year transition probability** | **Estimated 1-year transition probability** |
| High radiotherapy exposure (3.6 Gy) – smoker | 0.7129 | 0.1173 |
| Moderate radiotherapy exposure (1.9 Gy) – smoker | 0.6907 | 0.1107 |
| No radiotherapy exposure (0 Gy) – smoker | 0.6659 | 0.1038 |

| **SCORE morbidity prediction : Transition probability from initial state to non-fatal CV event** Assumption: calculated relative risk ratio for non-fatal CV events/fatal CV events in breast cancer survivors is 2.7515 *Calculation*^[[2]](#endnote-2)^ *rate of fatal CV events in a cohort of Dutch breast cancer survivors (n = 7,424): 2,411 (0.3248)*  *conversion to annual probability: 1-^(-(-(log(1-0.3248))/9)*1) = 0.0427 rate of non-fatal CV events in a cohort of Dutch breast cancer survivors (n = 7,424): 5,013 (0.6752)*  *conversion to annual probability: 1-^(-(-(log(1-0.6752))/9)*1) = 0.1175*  *Relative risk ratio: 0.0427 / 0.1175 = 2.7515* | | |
| --- | --- | --- |
| Age cohort: **40-49 year – non-smoker** | **Estimated 10-year transition probability** | **Estimated 1-year transition probability** |
| High radiotherapy exposure (3.6 Gy) – non-smoker | 0.0011 | 0.0001 |
| Moderate radiotherapy exposure (1.9 Gy) – non smoker | 0.0011 | 0.0001 |
| No radiotherapy exposure (0 Gy) – non smoker | 0.0010 | 0.0001 |
| Age cohort: **40-49 year – smoker** | **Estimated 10-year transition probability** | **Estimated 1-year transition probability** |
| High radiotherapy exposure (3.6 Gy) – smoker | 0.0022 | 0.0002 |
| Moderate radiotherapy exposure (1.9 Gy) – smoker | 0.0021 | 0.0002 |
| No radiotherapy exposure (0 Gy) – smoker | 0.0020 | 0.0002 |
| Age cohort: **50-59 year – non-smoker** | **Estimated 10-year transition probability** | **Estimated 1-year transition probability** |
| High radiotherapy exposure (3.6 Gy) – non-smoker | 0.0065 | 0.0007 |
| Moderate radiotherapy exposure (1.9 Gy) – non smoker | 0.0063 | 0.0006 |
| No radiotherapy exposure (0 Gy) – non smoker | 0.0061 | 0.0006 |
| Age cohort: **50-59 year – smoker** | **Estimated 10-year transition probability** | **Estimated 1-year transition probability** |
| High radiotherapy exposure (3.6 Gy) – smoker | 0.0128 | 0.0013 |
| Moderate radiotherapy exposure (1.9 Gy) – smoker | 0.0124 | 0.0012 |
| No radiotherapy exposure (0 Gy) – smoker | 0.0119 | 0.0012 |

| Age cohort: **60-69 year – non-smoker** | **Estimated 10-year transition probability** | **Estimated 1-year transition probability** |
| --- | --- | --- |
| High radiotherapy exposure (3.6 Gy) – non-smoker | 0.0250 | 0.0025 |
| Moderate radiotherapy exposure (1.9 Gy) – non smoker | 0.0242 | 0.0024 |
| No radiotherapy exposure (0 Gy) – non smoker | 0.0233 | 0.0024 |
| Age cohort: **60-69 year – smoker** | **Estimated 10-year transition probability** | **Estimated 1-year transition probability** |
| High radiotherapy exposure (3.6 Gy) – smoker | 0.0489 | 0.0050 |
| Moderate radiotherapy exposure (1.9 Gy) – smoker | 0.0473 | 0.0048 |
| No radiotherapy exposure (0 Gy) – smoker | 0.0454 | 0.0046 |
| Age cohort: **70-79 year – non-smoker** | **Estimated 10-year transition probability** | **Estimated 1-year transition probability** |
| High radiotherapy exposure (3.6 Gy) – non-smoker | 0.1130 | 0.0119 |
| Moderate radiotherapy exposure (1.9 Gy) – non smoker | 0.1094 | 0.0115 |
| No radiotherapy exposure (0 Gy) – non smoker | 0.1055 | 0.0111 |
| Age cohort: **70-79 year – smoker** | **Estimated 10-year transition probability** | **Estimated 1-year transition probability** |
| High radiotherapy exposure (3.6 Gy) – smoker | 0.2192 | 0.0244 |
| Moderate radiotherapy exposure (1.9 Gy) – smoker | 0.2121 | 0.0236 |
| No radiotherapy exposure (0 Gy) – smoker | 0.2041 | 0.0226 |
| Age cohort: **80-89 year – non-smoker** | **Estimated 10-year transition probability** | **Estimated 1-year transition probability** |
| High radiotherapy exposure (3.6 Gy) – non-smoker | 0.2759 | 0.0318 |
| Moderate radiotherapy exposure (1.9 Gy) – non smoker | 0.2675 | 0.0306 |
| No radiotherapy exposure (0 Gy) – non smoker | 0.2580 | 0.0294 |

| Age cohort: **80-89 year – smoker** | **Estimated 10-year transition probability** | **Estimated 1-year transition probability** |
| --- | --- | --- |
| High radiotherapy exposure (3.6 Gy) – smoker | 0.5278 | 0.0723 |
| Moderate radiotherapy exposure (1.9 Gy) – smoker | 0.5110 | 0.0690 |
| No radiotherapy exposure (0 Gy) – smoker | 0.4922 | 0.0655 |
| Age cohort: **90-99 year – non-smoker** | **Estimated 10-year transition probability** | **Estimated 1-year transition probability** |
| High radiotherapy exposure (3.6 Gy) – non-smoker | 0.5851 | 0.0842 |
| Moderate radiotherapy exposure (1.9 Gy) – non smoker | 0.5674 | 0.0804 |
| No radiotherapy exposure (0 Gy) – non smoker | 0.5477 | 0.0763 |
| Age cohort: **90-99 year – smoker** | **Estimated 10-year transition probability** | **Estimated 1-year transition probability** |
| High radiotherapy exposure (3.6 Gy) – smoker | 1.0000 | 0.9684 |
| Moderate radiotherapy exposure (1.9 Gy) – smoker | 1.0000 | 0.9684 |
| No radiotherapy exposure (0 Gy) – smoker | 1.0000 | 0.9684 |
| Age cohort: **100+ year – non-smoker** | **Estimated 10-year transition probability** | **Estimated 1-year transition probability** |
| High radiotherapy exposure (3.6 Gy) – non-smoker | 1.0000 | 0.9684 |
| Moderate radiotherapy exposure (1.9 Gy) – non smoker | 1.0000 | 0.9684 |
| No radiotherapy exposure (0 Gy) – non smoker | 1.0000 | 0.9684 |
| Age cohort: **100+ year – smoker** | **Estimated 10-year transition probability** | **Estimated 1-year transition probability** |
| High radiotherapy exposure (3.6 Gy) – smoker | 1.0000 | 0.9684 |
| Moderate radiotherapy exposure (1.9 Gy) – smoker | 1.0000 | 0.9684 |
| No radiotherapy exposure (0 Gy) – smoker | 1.0000 | 0.9684 |

1. Conroy RM, Pyörälä K, Fitzgerald AP, Sans S, Menotti A, De Backer G, et al. Estimation of ten-year risk of fatal cardiovascular disease in Europe: The SCORE project. Eur Heart J. 2003;24(11):987–1003. doi: 10.1016/S0195-668X(03)00114-3. [↑](#endnote-ref-1)
2. Boekel NB, Schaapveld M, Gietema JA, Russell NS, Poortmans P, Theuws JCM, et al. Cardiovascular disease risk in a large, population-based cohort of breast cancer survivors. Int J Radiat Oncol Biol Phys. 2016;94(5):1061–72. doi: 10.1016/j.ijrobp.2015.11.040. [↑](#endnote-ref-2)
